# Multidrug-resistant organisms on in-use medical textiles in a Shanghai tertiary hospital: surveillance and genomic characterization

**DOI:** 10.3389/fpubh.2026.1891356

**Published:** 2026-07-13

**Authors:** Chunyan Li, Jing Zhang, Lulu Gao, Yue Li, Yilun Zhou, Bin Wang, Na Ji, Shan Wang, Fei Wang, Liang Tian

**Affiliations:** 1 Disinfection and Vector Control Department, Hongkou District Center for Disease Control and Prevention (Hongkou District Institute of Health Supervision), Shanghai, China; 2 Microbiology Testing Division, Hongkou District Center for Disease Control and Prevention (Hongkou District Institute of Health Supervision), Shanghai, China; 3 Hospital Infection Control Department, Shanghai University of TCM Shanghai TCM-Integrated Hospital, Shanghai, China; 4 Department of Acute Communicable Diseases Control and Prevention, Hongkou District Center for Disease Control and Prevention (Hongkou District Institute of Health Supervision), Shanghai, China; 5 Department of Disinfection and Infection Control, Shanghai Municipal Center for Disease Control and Prevention, Shanghai, China

**Keywords:** CRAB, CRKP, infection prevention, medical textiles, MRSA, multidrug-resistant organisms

## Abstract

**Background:**

Medical textiles are frequently handled in healthcare settings and may serve as reservoirs for multidrug-resistant organisms (MDROs). This study investigated bacterial contamination, MDRO prevalence and genomic characteristics of MDRO isolates recovered from medical textiles in a tertiary hospital.

**Methods:**

A cross-sectional study was conducted between April and August 2024. Medical textile samples were collected from the outpatient department, general wards and intensive care unit (ICU). Bacterial contamination and bacterial load were assessed by microbiological culture, organism identification and antimicrobial susceptibility testing. MDRO isolates were further characterized using multilocus sequence typing, core-SNP phylogenetic analysis, antimicrobial resistance gene detection and virulence gene analysis.

**Results:**

A total of 359 samples from 21 types of medical textiles were collected. The overall MDRO detection rate was 8.64%, with the ICU showing the highest rate compared with general wards and outpatient departments. CRAB, MRSA, and CRKP were detected in 5.57, 2.23, and 0.84% of samples, respectively. Multivariable logistic regression showed that outpatient department sampling was associated with lower odds of MRSA detection (OR = 0.05, 95% CI: 0.01–0.48; *p* = 0.01), while airway opening was associated with lower odds of CRAB detection (OR = 0.21, 95% CI: 0.05–0.89; *p* = 0.03).

**Conclusion:**

By integrating environmental surveillance of in-use hospital textiles with genomic characterization of MDRO isolates, this study provides evidence for risk-based textile management and infection prevention strategies.

## Introduction

Healthcare-associated infections (HAIs) caused by multidrug-resistant organisms (MDROs) remain a major challenge for infection prevention and antimicrobial resistance control (1). The hospital environment can act as a reservoir for clinically important pathogens, particularly in high-risk areas such as intensive care units (ICUs), where patients are frequently exposed to invasive procedures, prolonged hospitalization and repeated contact with healthcare workers and environmental surfaces. Several bacterial pathogens relevant to this study, including carbapenem-resistant *Acinetobacter baumannii*, carbapenem-resistant *Klebsiella pneumoniae* and methicillin-resistant *Staphylococcus aureus*, are recognized as important antimicrobial-resistant pathogens of public health concern (2).

Medical textiles are widely used in healthcare settings and include bed sheets, patient gowns, staff uniforms, surgical textiles, towels, privacy curtains and cleaning textiles. These items are frequently handled, repeatedly reused and may come into direct or indirect contact with patients, healthcare workers and contaminated hospital environments. Previous reviews have suggested that healthcare textiles can retain microorganisms and may contribute to microbial transfer under certain conditions, although the magnitude of their contribution to MDRO transmission remains incompletely defined (3, 4).

Unlike hard environmental surfaces, medical textiles are soft, porous and often mobile. Their contamination may be influenced by textile material, moisture, frequency of use, patient contact, handling during collection and transport, and the effectiveness of laundering and storage procedures. Studies have shown that healthcare textiles and apparel may be contaminated with clinically relevant microorganisms, and that some healthcare-associated pathogens can persist on hospital fabrics for prolonged periods, especially under moist conditions (5, 6). These findings indicate that medical textiles should be considered in environmental hygiene and infection prevention programmes.

In China, technical standards for the washing and disinfection of medical textiles have been established. However, in routine practice, medical textile management may involve multiple steps, including bedside collection, classification, transport, outsourced laundering, storage and redistribution. Each step may represent a potential point for contamination or recontamination if infection prevention procedures are not strictly implemented. Nevertheless, data on MDRO contamination of medical textiles in Chinese hospitals remain limited, and few studies have combined bacterial load assessment, MDRO detection and genomic characterization of textile-associated isolates (7).

Whole-genome sequencing and related genomic approaches provide higher resolution for characterizing MDRO isolates than conventional microbiological methods alone (8). These approaches can be used to determine sequence types, identify antimicrobial resistance and virulence genes, and assess genetic relatedness among isolates. Genomic surveillance has increasingly been used to support hospital epidemiology and infection prevention, particularly for investigating potential transmission of antimicrobial-resistant pathogens (9, 10).

Therefore, this study aimed to investigate bacterial contamination and MDRO contamination of medical textiles collected from different hospital departments in a tertiary hospital. Unlike previous studies that mainly focused on bacterial load or culture-based contamination, our study combined environmental textile surveillance with genomic characterization, including MLST, core-SNP phylogenetic analysis, antimicrobial resistance gene profiling, and virulence gene analysis, to better understand the potential role of medical textiles in MDRO dissemination.

## Materials and methods

### Study design and setting

This cross-sectional study was conducted in a tertiary comprehensive hospital in Shanghai, China, between April and August 2024. Medical textile samples were collected from three hospital areas: the outpatient department, general wards and the intensive care unit (ICU). Stratified random sampling was used in this study. The sampling areas were first stratified into general wards, ICU, and outpatient department. Within each hospital area, eligible textiles were further stratified according to 21 categories of medical textiles. All eligible textile items within each stratum were numbered, and samples were selected using a random number method. No manual selection was performed during sampling, thereby minimizing potential sampling bias.

The sampled medical textiles included patient bed sheets, quilt covers, patient gowns, healthcare workers’ uniforms, surgical gowns, towels, floor towels, cleaning cloths, privacy curtains and other reusable textile items used in clinical practice. A total of 359 samples from 21 types of medical textiles were included in the analysis.

### Sample collection

Samples were collected under aseptic conditions. For each textile item, both inner and outer surfaces were exposed when applicable. A sterile 5 cm × 5 cm template was placed on the sampling area. A sterile swab pre-moistened with sterile saline was used to swab the marked area horizontally and vertically five times in each direction while rotating the swab. Four non-overlapping areas were sampled from each textile item, covering a total area of 100 cm^2^. The limit of detection of the culture-based plating method was 10 CFU/100 cm^2^.

After sampling, the swab head was placed into a sterile tube containing 10 mL of transport solution. All samples were transported to the laboratory and processed within 4 h after collection.

### Assessment of textile condition and bacterial load

The visual condition of each textile was assessed before microbiological testing, including cleanliness, dryness, odour, visible foreign matter and visible damage. Visible blood or bodily fluid contamination was also recorded.

Total bacterial colony counts were determined and expressed as colony-forming units per 100 cm^2^ (CFU/100 cm^2^). According to the relevant Chinese standards (WS/T 508-2025 and GB 15982-2012) (11, 12) for hospital textile washing and healthcare disinfection, two bacterial load thresholds were used in this study: >10 CFU/100 cm^2^ and >200 CFU/100 cm^2^. Samples exceeding these thresholds were classified as having low-level and high-level bacterial contamination, respectively.

Medical textiles were managed through standardized procedures covering collection, transport, laundering and disinfection, storage, and redistribution. Contaminated textiles were classified as general or infectious textiles, sealed separately, and transported using dedicated enclosed vehicles through designated contaminated-material routes. Transport tools for clean and contaminated textiles were strictly separated, and the temporary storage time for contaminated textiles did not exceed 48 h. Laundering and disinfection were performed in accordance with WS/T 508-2025, mainly using high-temperature thermal disinfection supplemented by chemical disinfection when necessary. The laundering process included pre-washing, main washing, rinsing, neutralization, drying, and ironing. Clean textiles that met quality requirements were stored in an independent, well-ventilated clean storage room, managed by category, and subjected to regular environmental disinfection. Clean textiles were redistributed within the hospital using dedicated transport vehicles to minimize cross-contamination and secondary contamination.

### Isolation and identification of target bacteria

After sample collection, tubes were allowed to stand at room temperature for 15 min. The sample suspension was mixed thoroughly, and aliquots were inoculated onto selective or differential culture media. *Staphylococcus aureus* was cultured on blood agar plates, whereas *A. baumannii* and *K. pneumoniae* were cultured on MacConkey agar plates. Plates were incubated at 35 °C for 24 h. To avoid overrepresentation of duplicate isolates, only one representative isolate of the same species and colony morphology from the same textile item was included for downstream analysis. Distinct colony morphologies or different species from the same item were analyzed separately.

Suspected colonies were selected based on colony morphology and subcultured to obtain pure isolates. Purified isolates were identified using matrix-assisted laser desorption/ionization time-of-flight mass spectrometry with the Autof MS1000 system. Isolates meeting the manufacturer-recommended identification score threshold were confirmed as *A. baumannii*, *K. pneumoniae* or *S. aureus*.

*Acinetobacter baumannii*, *K. pneumoniae*, and *S. aureus* were selected as target organisms because CRAB, CRKP, and MRSA are key MDROs under routine hospital infection surveillance and represent clinically important Gram-negative and Gram-positive pathogens with well-documented environmental persistence and transmission potential. We acknowledge that other healthcare-associated pathogens, such as vancomycin-resistant *Enterococcus* and *Pseudomonas aeruginosa*, were not included, which may have underestimated the overall MDRO burden on medical textiles.

### Antimicrobial susceptibility testing and MDRO definition

Antimicrobial susceptibility testing was performed using the WalkAway 96 PLUS automated microbial identification and susceptibility testing system. Results were interpreted according to the Clinical and Laboratory Standards Institute M100 guidelines. Quality control strains included *A. baumannii* ATCC 19606, *K. pneumoniae* ATCC 700603 and *S. aureus* ATCC 25923.

In this study, MDROs included carbapenem-resistant *A. baumannii* (CRAB), carbapenem-resistant *K. pneumoniae* (CRKP) and methicillin-resistant *S. aureus* (MRSA). CRAB and CRKP were defined as isolates resistant to at least one carbapenem agent tested. MRSA was defined as *S. aureus* resistant to oxacillin or cefoxitin, or carrying the mecA gene. Isolates resistant to one or more agents in at least three antimicrobial classes were also considered multidrug-resistant.

For CRAB isolates, the antimicrobial panel included 18 agents: ampicillin/sulbactam, ticarcillin/clavulanic acid, piperacillin, piperacillin/tazobactam, ceftriaxone, ceftazidime, cefotaxime, cefepime, meropenem, imipenem, ciprofloxacin, levofloxacin, amikacin, gentamicin, tobramycin, trimethoprim/sulfamethoxazole, tetracycline, and tigecycline. For MRSA isolates, the antimicrobial panel included 16 agents: chloramphenicol, clindamycin, ciprofloxacin, daptomycin, erythromycin, gentamicin, levofloxacin, linezolid, moxifloxacin, oxacillin, penicillin, rifampicin, quinupristin/dalfopristin, trimethoprim/sulfamethoxazole, tetracycline, and vancomycin, together with a cefoxitin screening test. For CRKP isolates, the antimicrobial panel included 22 agents: ampicillin/sulbactam, amikacin, aztreonam, ceftriaxone, ceftazidime, cefotaxime, cefoxitin, cefazolin, ciprofloxacin, cefepime, cefuroxime, gentamicin, imipenem, levofloxacin, meropenem, piperacillin/tazobactam, piperacillin, trimethoprim/sulfamethoxazole, tetracycline, tigecycline, ticarcillin/clavulanic acid, and tobramycin.

### Collection of potential influencing factors

Potential factors associated with MDRO contamination were recorded for each textile sample. These included hospital department, textile type, frequency of textile replacement, washing method, sampling time after textile use, presence of visible blood or bodily fluid contamination, presence of an artificial airway in the corresponding patient, patient diarrhoea, bedridden status and direct contact with patients known to carry MDROs. These variables were used to explore potential associations with MRSA, CRAB and CRKP detection.

### Whole-genome sequencing

All MDRO isolates, including CRAB, CRKP and MRSA isolates, were subjected to whole-genome sequencing. Genomic DNA was extracted using a commercial DNA extraction kit according to the manufacturer’s instructions. DNA concentration and quality were assessed before library preparation.

Whole-genome sequencing was performed using the Illumina NovaSeq X Plus platform. The average sequencing depths were 335.1325× for *S. aureus*, 272.0520× for *A. baumannii*, and 175.6094× for *K. pneumoniae*. Sequencing libraries were prepared and sequenced on an Illumina high-throughput sequencing platform by Shanghai Zhongke Runda Medical Laboratory Co., Ltd. Raw reads were filtered using fastp to remove low-quality reads and adaptor sequences.

### Genomic analysis

High-quality reads were used for genomic analysis. Multilocus sequence typing was performed using the PubMLST database to determine sequence types. Antimicrobial resistance genes were identified using AMRFinderPlus. Virulence-associated genes were detected using the Virulence Factor Database.

Core single-nucleotide polymorphisms were used to assess the genetic relatedness of isolates within the same species. Phylogenetic trees were constructed based on core-SNP alignments. Isolates with small core-SNP differences were considered genetically closely related, and potential clusters were interpreted together with sampling date, department and textile type.

After quality control, high-quality reads were assembled *de novo* using EToKi. Assembly quality was evaluated based on total genome length, GC content, N50, N90, and the number of contigs before downstream analyses, including MLST, antimicrobial resistance gene profiling, virulence gene detection, and core-SNP phylogenetic analysis.

### Statistical analysis

Data were managed using Microsoft Excel 2019 and analysed using SPSS version 21.0. Categorical variables were presented as frequencies and percentages. Differences in bacterial detection rates, MDRO detection rates and bacterial load categories among departments or textile types were compared using the chi-square test or Fisher’s exact test, as appropriate.

Univariate analyses were performed to explore factors associated with MRSA, CRAB and CRKP detection. Variables with statistical significance in univariate analysis and variables considered clinically relevant were considered for multivariable logistic regression. Before multivariable logistic regression, multicollinearity among candidate variables was assessed using variance inflation factors and tolerance values. Variables with high collinearity were not included simultaneously in the same model. Odds ratios and 95% confidence intervals (CIs) were reported. A two-sided *p* value <0.05 was considered statistically significant.

## Results

### Bacterial contamination and MDRO detection by department

A total of 359 medical textile samples were collected from the outpatient department, general wards and ICU (Table 1). Overall, 112 samples were positive for *A. baumannii*, *S. aureus* or *K. pneumoniae*, giving a bacterial detection rate of 31.20%. The detection rate differed significantly among departments, with the highest rate observed in general wards (52/120, 43.33%), followed by the ICU (40/120, 33.33%) and outpatient department (20/119, 16.81%) (*χ*^2^ = 19.97, *p* < 0.001).

**Table 1:** Bacterial load and multidrug-resistant organism (MDRO) contamination by hospital department.

Department	No. of samples	AB/SA/KP-positive, *n*	Detection rate (%)	*χ*², *p* value	MDRO-positive, *n*	MDRO detection rate (%)	*χ*², *p* value	>10 CFU/100 cm², *n*	Detection rate (%)	*χ*², *p* value	>200 CFU/100 cm², n	Detection rate (%)	*χ*², *p* value
Outpatient department	119	20	16.81%	19.97, <0.001	2	1.68%	17.30, <0.001	33	27.73%	9.57, 0.009	4	3.36%	5.84, 0.054
General ward	120	52	43.33%		9	7.50%		55	45.83%		14	11.67%	
ICU	120	40	33.33%		20	16.67%		38	31.67%		11	9.17%	
Total	359	112	31.20%	—	31	8.64%	—	126	35.10%	—	29	8.08%	—

AB, *Acinetobacter baumannii*; SA, *Staphylococcus aureus*; KP, *Klebsiella pneumoniae*.

MDROs were detected in 31 samples, with an overall detection rate of 8.64%. The ICU had the highest MDRO detection rate (20/120, 16.67%), followed by general wards (9/120, 7.50%) and the outpatient department (2/119, 1.68%) (*χ*^2^ = 17.30, *p* < 0.001) (Table 1).

A total of 126 samples (35.10%) had bacterial colony counts >10 CFU/100 cm^2^. The proportion was highest in general wards (55/120, 45.83%), followed by the ICU (38/120, 31.67%) and outpatient department (33/119, 27.73%) (*χ*^2^ = 9.57, *p* = 0.009). Colony counts >200 CFU/100 cm^2^ were identified in 29 samples (8.08%), with no statistically significant difference among departments (*p* = 0.054) (Table 1).

### Distribution of MDROs across textile types

Among the 359 samples, CRAB was the most frequently detected MDRO, with 20 positive samples (5.57%). MRSA was detected in 8 samples (2.23%), and CRKP was detected in 3 samples (0.84%) (Table 2).

**Table 2:** Bacterial load and multidrug-resistant organism (MDRO) contamination by medical textile type.

Textile type	No. of samples	CRAB-positive, *n*	CRAB detection rate (%)	MRSA-positive, *n*	MRSA detection rate (%)	CRKP-positive, *n*	CRKP detection rate (%)	>10 CFU/100 cm², *n*	Detection rate (%)	>200 CFU/100 cm², *n*	Detection rate (%)
Isolation gown	5	1	20.00%	0	0.00%	0	0.00%	3	60.00%	0	0.00%
Oxygen bag	5	1	20.00%	0	0.00%	0	0.00%	2	40.00%	0	0.00%
Fabric bag	12	0	0.00%	2	16.67%	0	0.00%	4	33.33%	1	8.33%
Curtain	6	0	0.00%	0	0.00%	0	0.00%	0	0.00%	0	0.00%
Towel	6	0	0.00%	0	0.00%	0	0.00%	3	50.00%	1	16.67%
Shower curtain	6	0	0.00%	0	0.00%	0	0.00%	3	50.00%	2	33.33%
Pillow and bedding	30	3	10.00%	2	6.67%	2	6.67%	19	63.33%	4	13.33%
Work clothes	58	5	8.62%	2	3.45%	0	0.00%	17	29.31%	0	0.00%
Patient gown	36	4	11.11%	1	2.78%	0	0.00%	22	61.11%	6	16.67%
Privacy curtain	78	3	5.08%	0	0.00%	1	1.69%	11	14.10%	1	1.28%
Floor towel	12	1	8.33%	0	0.00%	0	0.00%	8	66.67%	5	41.67%
Cotton mattress pad	8	0	0.00%	0	0.00%	0	0.00%	3	37.50%	0	0.00%
Pressure pillow	8	1	12.50%	0	0.00%	0	0.00%	2	25.00%	0	0.00%
Bed sheet	35	0	0.00%	0	0.00%	0	0.00%	16	45.71%	7	20.00%
Hat	16	0	0.00%	1	6.25%	0	0.00%	1	6.25%	0	0.00%
Mattress	8	0	0.00%	0	0.00%	0	0.00%	2	25.00%	0	0.00%
Cover cloth	16	0	0.00%	0	0.00%	0	0.00%	1	6.25%	0	0.00%
Blanket sheet	2	0	0.00%	0	0.00%	0	0.00%	0	0.00%	0	0.00%
Pillow	8	0	0.00%	0	0.00%	0	0.00%	6	75.00%	0	0.00%
Rag	4	1	25.00%	0	0.00%	0	0.00%	3	75.00%	2	50.00%
Total	359	20	5.57%	8	2.23%	3	0.84%	126	35.10%	29	8.08%

CRAB was mainly detected from rags, isolation gowns, oxygen bags, patient gowns, pillow and bedding items, work clothes, privacy curtains and floor towels. The highest CRAB detection rates were observed in rags (1/4, 25.00%), isolation gowns (1/5, 20.00%) and oxygen bags (1/5, 20.00%) (Table 2).

MRSA was detected in fabric bags, pillow and bedding items, work clothes, patient gowns and hats. The highest MRSA detection rate was found in fabric bags (2/12, 16.67%). CRKP was detected only in pillow and bedding items and privacy curtains.

Regarding bacterial load, colony counts >10 CFU/100 cm^2^ were most frequently observed in rags and pillows, followed by floor towels, pillow and bedding items, patient gowns and isolation gowns. Colony counts >200 CFU/100 cm^2^ were most common in rags, floor towels and shower curtains.

### Factors associated with MDRO detection

In univariate analysis, MRSA detection was associated with visible blood or bodily fluid contamination (*p* = 0.004) (Table 3). CRAB detection was associated with department, airway-opening status, bedridden status and direct contact with patients carrying MDROs (all *p* < 0.05). CRKP detection was associated with airway-opening status and bedridden status (both *p* < 0.05).

**Table 3:** Univariate analysis of factors associated with MDRO contamination of medical textiles.

Category		MRSA				CRAB				CRKP			
		Positive, *n*	Negative, *n*	*χ*²	*p* value	Positive, *n*	Negative, *n*	*χ*²	*p* value	Positive, *n*	Negative, *n*	*χ*²	*p* value
Department	Outpatient	1	118	3.22	0.200*	1	118	7.156	<0.001*	0	119	4.048	0.109**
	General ward	5	115			4	116			0	120		
	ICU	2	118			15	105			3	117		
Fabric change frequency	Once a day	0	78	4.073	0.455**	3	75	8.418	0.098**	0	78	3.55	0.629**
	Twice a week	2	51			2	51			0	53		
	Once a week	3	74			10	67			2	75		
	Once every two weeks	0	17			0	17			0	17		
	Once a month	3	98			3	98			1	100		
	Not replaced	0	33			2	31			0	33		
Washing method	Not disinfected	0	61	1.479	0.472**	3	58	0.061	0.970*	0	61	0.429	1**
	Self-disinfecting	1	34			2	33			0	35		
	Outsourced	7	256			15	248			3	260		
Sampling time (d)	0 d	0	75	4.106	0.4250*	2	73	1.91	0.591*	0	75	2.338	0.485**
	1–5 d (including 1)	5	146			9	142			2	149		
	5–14 d (including 5)	2	82			6	78			0	84		
	≥14 d	1	48			3	46			1	48		
Obvious blood or bodily fluid contamination	No	5	337	—	0.004**	20	322	—	0.612**	3	339	—	1**
	Yes	3	14			0	17			0	17		
Airway opening	No	7	260	0.203	0.652*	6	261	21.88	<0.001	0	267	—	0.016**
	Yes	1	91			14	78			3	89		
Patient diarrhoea	No	5	312	—	0.055**	17	300	0.013	0.909*	3	314	—	1**
	Yes	3	39			3	39			0	42		
Patient bedridden	No	4	259	2.001	0.157*	8	255	11.959	0.01	0	263	—	0.019**
	Yes	4	92			12	84			3	93		
Direct contact with MDROs patients	No	3	250	2.808	0.094*	9	244	6.605	0.01	1	252	—	0.209**
	Yes	5	101			11	95			2	104		
Total colony count	0	2	94	1.56	0.672**	7	89	4.903	0.179*	1	95	3.047	0.432**
	0–10 (including 10)	2	136			6	132			0	138		
	10–200 (including 200)	3	93			3	93			2	94		
	>200	1	28			4	25			0	29		

^*^p value calculated using the likelihood-ratio test; ^**^p value calculated using Fisher’s exact test.

In multivariable analysis, department remained significantly associated with CRAB detection (overall *p* = 0.036) (Table 4). Absence of an open airway was also independently associated with CRAB detection (OR = 0.21, 95% CI: 0.05–0.89; *p* = 0.033). No statistically significant independent factor was identified for CRKP detection (Table 4).

**Table 4:** Multivariable logistic regression analysis of factors associated with MDRO contamination of medical textiles.

Variable		*β*	SE	Wald *χ*²	OR	95% CI	*p* value
MRSA	Blood and body fluid contamination	−2.855	0.464	37.926	0.058	0.023–0.143	
CRAB	Department			6.669	0.036		0.036
	Department (1)	−3.031	1.176	6.639	0.048	0.005–0.484	0.010
	Department (2)	0.699	0.701	0.996	0.497	0.126–1.963	0.318
	No open airway vs open airway	−1.570	0.739	4.520	0.208	0.049–0.885	0.033
	Bedridden	0.531	0.506	1.101	1.700	0.631–4.580	0.294
	Contact with multidrug-resistant patients	−0.075	0.334	0.051	0.928	0.482–1.784	0.822

### MLST typing

MLST analysis was performed for 31 MDRO isolates, including 8 MRSA, 20 CRAB and 3 CRKP isolates. Among MRSA isolates, ST398 was the predominant sequence type (5/8, 62.50%), followed by ST764 (2/8, 25.00%) and ST1 (1/8, 12.50%) (Table 5).

**Table 5:** Distribution of multilocus sequence typing (MLST) among MDRO isolates.

Organism	Sequence type	No. of isolates	Proportion (%)
MRSA	ST398	5	62.50%
	ST764	2	25.00%
	ST1	1	12.50%
CRAB	ST2	15	75.00%
	ST164	2	10.00%
	ST727	1	5.00%
	ST132	1	5.00%
	ST25	1	5.00%
CRKP	ST15	3	100.00%

Among CRAB isolates, ST2 was the most common sequence type (15/20, 75.00%). Other sequence types included ST164 (2/20, 10.00%), ST727 (1/20, 5.00%), ST132 (1/20, 5.00%) and ST25 (1/20, 5.00%) (Table 5). All CRKP isolates belonged to ST15.

### Phylogenetic analysis

**Figure 1:**
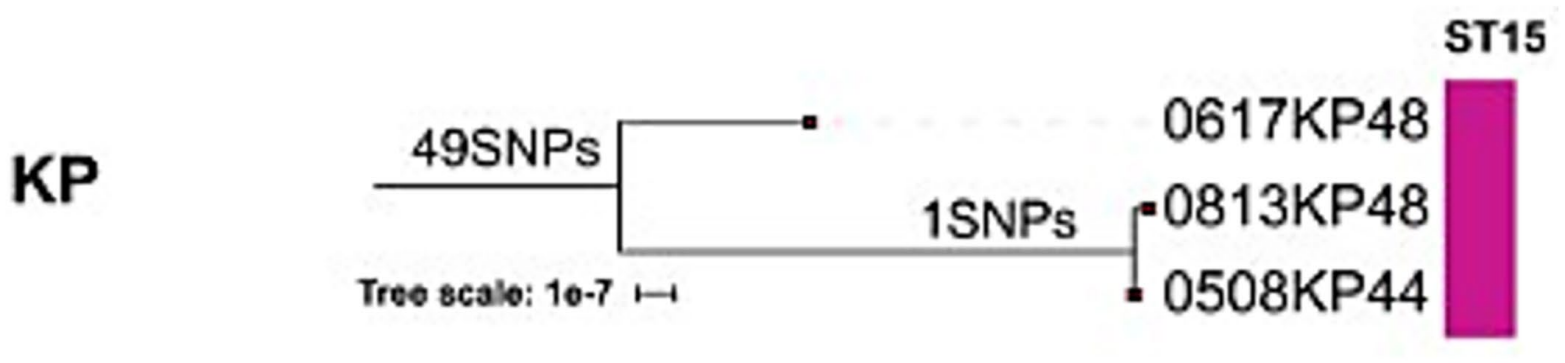
Core-SNP phylogeny of CRKP isolates recovered from medical textiles. Strain labels indicate isolate identifiers. A closely related pair differing by one SNP suggests possible common origin.

**Figure 2:**
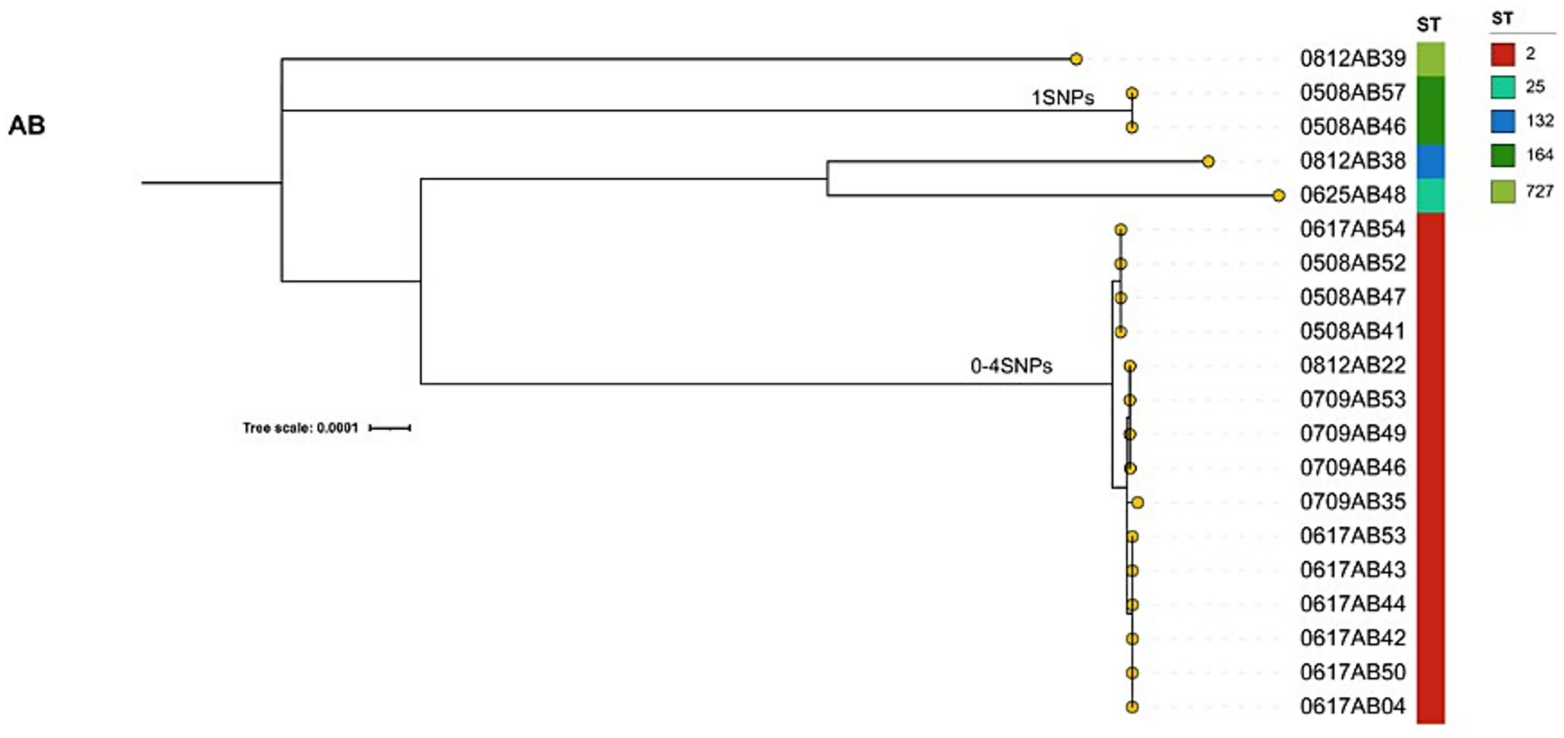
Core-SNP phylogeny of CRAB isolates recovered from medical textiles. Closely related clusters indicate possible circulation of related strains across textile categories or hospital areas.

**Figure 3:**
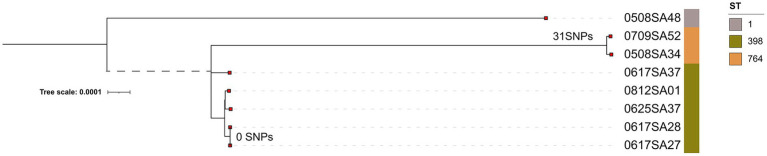
Core-SNP phylogeny of MRSA isolates recovered from medical textiles. Genetically close isolates suggest possible local dissemination within the textile environment.

Core-SNP phylogenetic analysis showed close genetic relatedness among selected isolates. Two CRKP isolates, 0813KP48 and 0508KP44 (Figure 1), differed by only one SNP, suggesting a close clonal relationship. Among CRAB isolates, two closely related clusters were identified, mainly involving ST164 and ST2 isolates (Figure 2). For MRSA, isolates 0617SA28 and 0617SA27 showed no core-SNP differences, indicating a highly similar genomic background (Figure 3).

### Antimicrobial resistance genes

**Figure 4:**
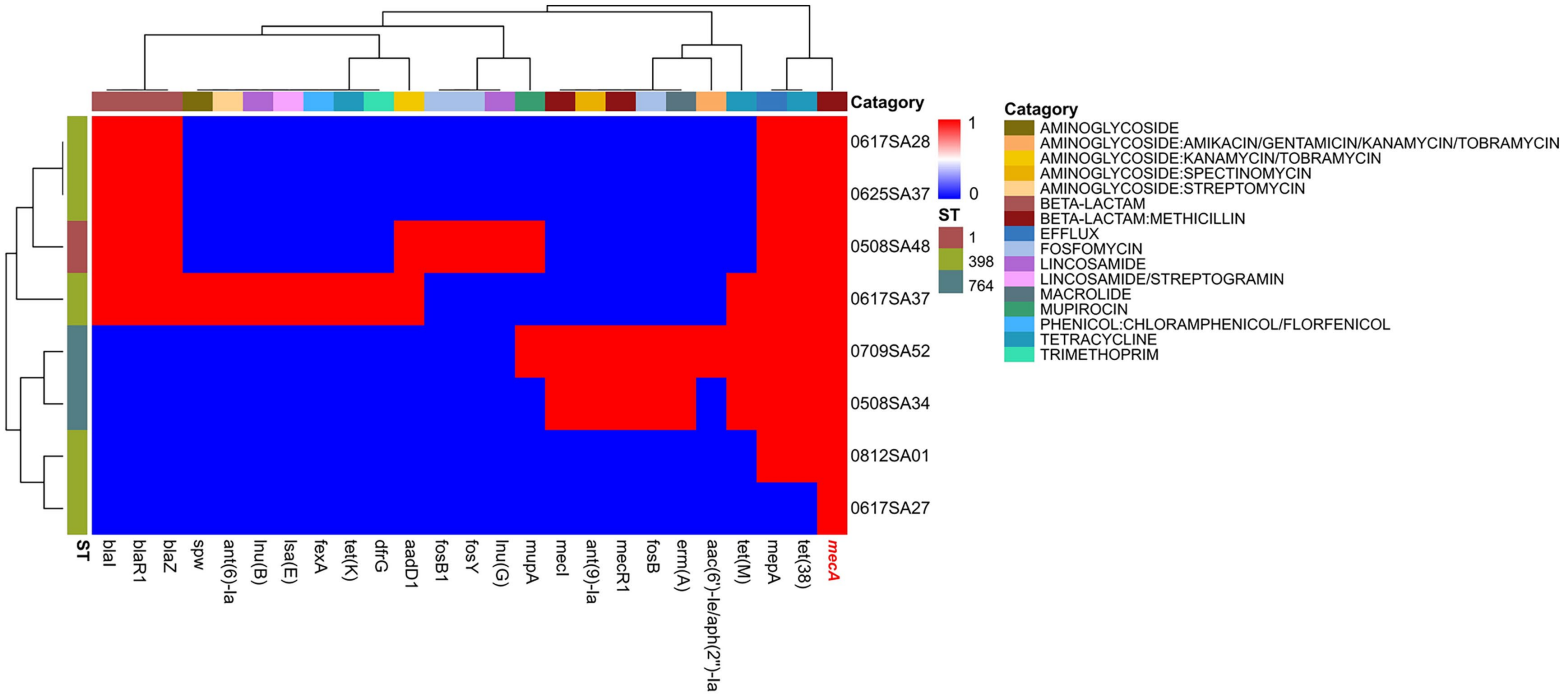
Heatmap of resistance genes detected in CRKP isolates. Color intensity indicates gene copy number or presence level; carbapenemase genes are highlighted in red.

All CRKP isolates carried blaKPC-2, together with multiple resistance genes related to macrolides, fosfomycin, efflux pumps and β-lactams. Isolates 0813KP48 and 0508KP44 showed identical antimicrobial resistance gene profiles (Figure 4).

**Figure 5:**
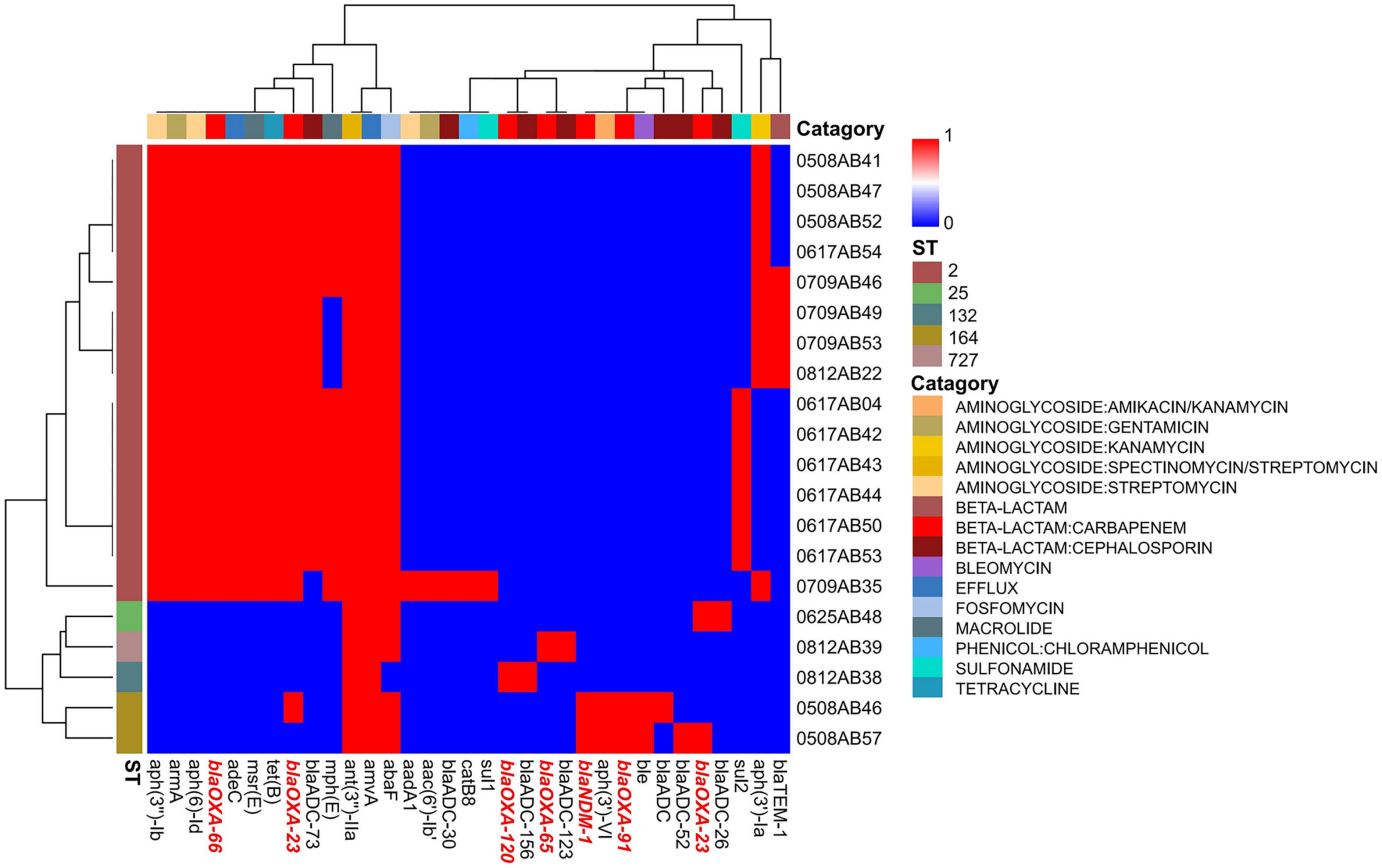
Heatmap of resistance genes detected in CRAB isolates. Color intensity indicates gene copy number or presence level; carbapenemase genes are highlighted in red.

Among CRAB isolates, ant(3″)-IIa and amvA were detected in all isolates. The fosfomycin resistance gene abaF was present in 95.00% of CRAB isolates. The most common carbapenemase genes were blaOXA-23 and blaOXA-66, detected in 80.00 and 75.00% of isolates, respectively. Several CRAB isolates shared identical resistance gene profiles, forming three major resistance gene clusters (Figure 5).

**Figure 6:**
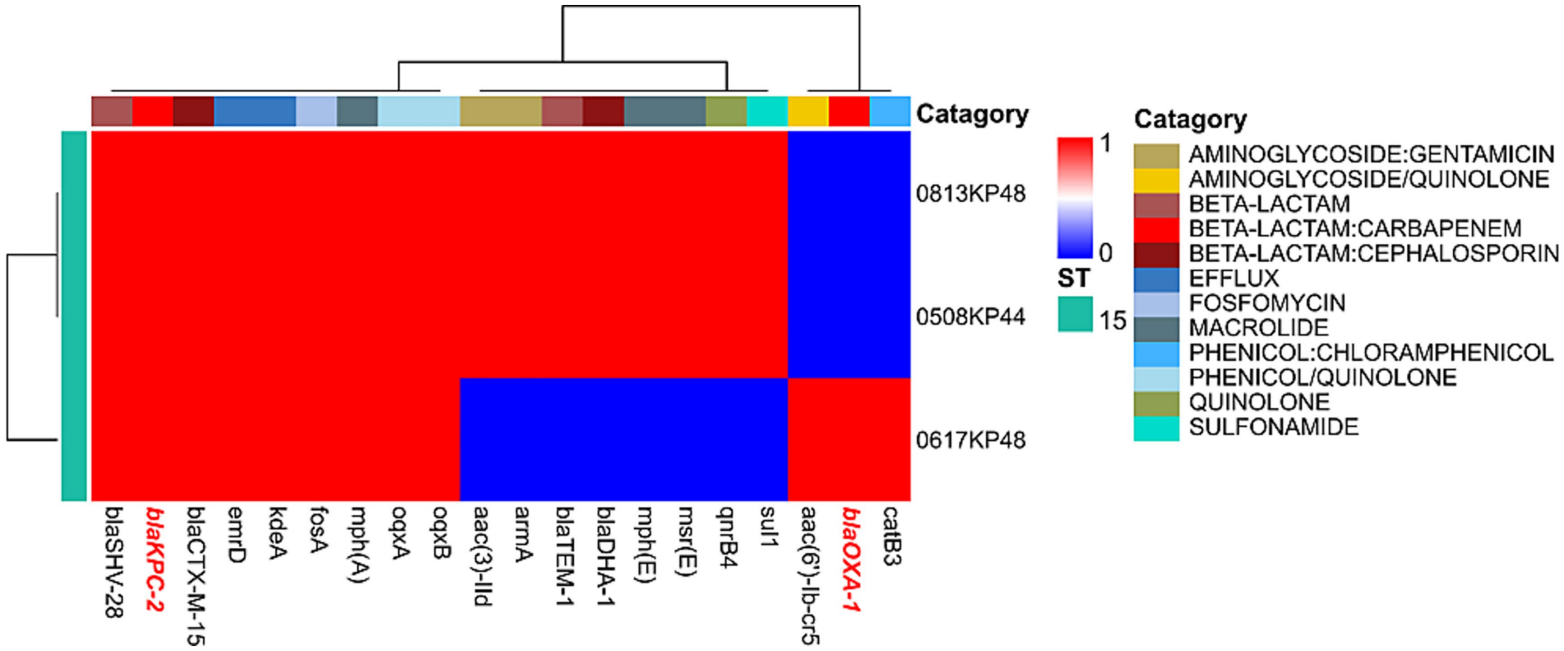
Heatmap of resistance genes detected in MRSA isolates. Color intensity indicates gene copy number or presence level.

All MRSA isolates carried mecA. The tet(38) and mepA genes were detected in 87.50% of MRSA isolates. The β-lactamase genes blaZ, blaR1 and blaI were each detected in 50.00% of isolates. Two MRSA isolates, 0617SA28 and 0625SA37, showed identical resistance gene profiles (Figure 6).

### Virulence genes

CRKP isolates carried virulence genes related to capsular polysaccharide synthesis, siderophore systems and efflux pumps. Two CRKP isolates showed virulence genes related to capsular polysaccharide synthesis, siderophore systems and efflux pumps. Two CRKP isolates showed identical virulence gene profiles.

CRAB isolates commonly carried genes associated with acinetobactin, AdeFGH efflux pumps and PbpG. Several CRAB isolates shared identical virulence gene profiles, consistent with the clustering observed in resistance gene analysis.

MRSA isolates carried virulence genes encoding thermonuclease, fibronectin-binding proteins and clumping factors. Isolates 0617SA28, 0625SA37 and 0812SA01 showed identical virulence gene profiles.

## Discussion

This cross-sectional study evaluated bacterial contamination and MDRO contamination of medical textiles in a tertiary hospital. Overall, MDROs were detected in 8.64% of medical textile samples, with the highest detection rate observed in the ICU. CRAB was the predominant MDRO, followed by MRSA and CRKP. In addition, WGS analysis revealed several genetically related isolates, suggesting that medical textiles may serve as potential environmental reservoirs for MDROs in hospital settings.

Medical textiles are increasingly recognized as potential fomites in healthcare environments. Bed linens, patient gowns, staff uniforms, privacy curtains and cleaning textiles are frequently touched, repeatedly used and often exposed to patients, healthcare workers and the surrounding environment. Previous reviews have shown that healthcare textiles can retain microorganisms and may contribute to microbial transfer under certain conditions, especially when moisture, repeated contact or friction is present (3, 4). In the present study, the detection of MDROs from multiple textile categories supports the need to include medical textiles in routine environmental hygiene and infection prevention programmes.

The higher MDRO detection rate in the ICU is consistent with the characteristics of high-risk clinical areas. ICU patients are more likely to have severe underlying disease, prolonged hospitalization, invasive devices and frequent contact with healthcare workers and environmental surfaces. Environmental contamination in ICUs has been recognized as an important component of MDRO transmission risk (13). In this study, the ICU showed the highest MDRO detection rate, particularly for CRAB, suggesting that textile management in high-risk wards should receive greater attention.

The distribution of contamination also differed by textile type. Cleaning textiles, floor towels, patient gowns, pillow and bedding items, and privacy curtains showed relatively high bacterial loads or MDRO detection. These textiles either have frequent patient contact or are used in environmental cleaning, both of which may increase the possibility of microbial acquisition and redistribution. Previous studies have shown that privacy curtains can become rapidly contaminated with potentially pathogenic bacteria, including MRSA and VRE (14). Similarly, healthcare workers’ uniforms have been reported to carry multidrug-resistant bacteria and may contribute to cross-contamination in clinical settings (15). These findings are consistent with our observation that both patient-contact textiles and cleaning-related textiles may represent important control points.

CRAB was the most frequently detected MDRO in this study. This finding is clinically relevant because *A. baumannii* can persist in hospital environments and is frequently associated with ICU outbreaks. The predominance of ST2 among CRAB isolates is consistent with the epidemiology of carbapenem-resistant *A. baumannii* in China and internationally. A nationwide genomic surveillance study in China reported that CRAB was prevalent in ICUs and that clonal spread was observed in a substantial proportion of hospitals (16). The frequent detection of blaOXA-23 and blaOXA-66 in our CRAB isolates is also consistent with the known molecular features of ST2 CRAB in China (17).

All CRKP isolates in this study belonged to ST15 and carried blaKPC-2. ST15 CRKP has been increasingly recognized as a high-risk lineage associated with hospital transmission. Previous studies have reported ST15 CRKP isolates co-producing KPC-2, CTX-M-15 and SHV-type β-lactamases in ICU settings (18). Although only three CRKP isolates were detected in the present study, the close SNP distance between two isolates from ICU textiles suggests a possible shared source or local cross-contamination. However, because no paired patient isolates or longitudinal environmental samples were available, the direction and route of transmission cannot be confirmed.

Among MRSA isolates, ST398 was the predominant sequence type, followed by ST764 and ST1. ST398 and ST764 have both been reported among MRSA isolates in China, including hospital and environmental sources. A WGS-based study from Shanghai found that ST764 and ST398 were important MRSA lineages and highlighted the need for ongoing surveillance of emerging MRSA clones (19). In the present study, two MRSA isolates showed no core-SNP differences, indicating a highly similar genomic background. This finding supports the possibility of local clonal contamination, but it should be interpreted cautiously because the study did not include patient screening or healthcare worker sampling.

The antimicrobial resistance gene profiles further support the clinical relevance of the textile-associated isolates. All MRSA isolates carried mecA, the major determinant of methicillin resistance. CRAB isolates frequently carried OXA-type carbapenemase genes and efflux pump-associated genes, while CRKP isolates carried blaKPC-2 together with additional β-lactam and efflux-related resistance genes. These resistance profiles indicate that MDROs recovered from medical textiles may carry clinically important resistance determinants. Nevertheless, the presence of resistance genes alone does not prove active transmission or infection risk; rather, it highlights the need for integrated environmental and genomic surveillance.

Virulence gene analysis showed that CRKP, CRAB and MRSA isolates carried genes associated with adhesion, iron acquisition, capsule formation, efflux systems or other pathogenic traits. For example, siderophore and capsule-associated genes are important contributors to *K. pneumoniae* pathogenicity, while acinetobactin and efflux systems contribute to the adaptation and fitness of *A. baumannii* in healthcare environments (20, 21). However, the present study only predicted virulence genes from genomic data. Functional assays, transcriptomic analysis or infection models would be required to determine whether these genes translate into enhanced virulence.

This study has several infection prevention implications. First, medical textiles should not be viewed only as laundry products, but also as potential environmental reservoirs that require risk-based management. Second, ICU textiles, cleaning textiles, privacy curtains, patient gowns, pillows and bedding items should be prioritized for monitoring and targeted disinfection. Third, the textile management process should be considered as a whole chain, including collection, transport, washing, drying, storage, distribution and ward-level reuse. Antimicrobial textiles, such as copper oxide-impregnated linens, have been explored as adjunctive interventions in healthcare settings, but they should complement rather than replace standard cleaning, disinfection and laundry procedures (22).

*Klebsiella pneumoniae* is a major opportunistic pathogen responsible for healthcare-associated infections, including pneumonia, bloodstream infections, urinary tract infections, and device-associated infections (23). The emergence of carbapenem-resistant *K. pneumoniae* has become a serious clinical challenge because treatment options are limited and outbreaks can occur in high-risk hospital settings (24). Molecular diagnostic approaches, including rapid resistance gene detection and whole-genome sequencing, are increasingly important for identifying carbapenemase genes, monitoring clonal dissemination, and guiding infection control responses. In addition to conventional antimicrobial therapy and infection prevention measures, emerging alternative strategies, including antimicrobial nanomaterials, nanoparticle-coated medical devices, phytochemical compounds, and biofilm-targeting interventions, have shown potential antibacterial activity against resistant *K. pneumoniae* (25). Although these approaches remain largely experimental or translational, they may provide complementary tools for reducing pathogen persistence on medical materials and healthcare surfaces.

The general wards showed the highest overall bacterial detection rate, whereas the ICU had the highest MDRO detection rate. This discrepancy may reflect different contamination mechanisms. General wards have high patient turnover, frequent textile use, and diverse routine care activities, which may increase overall bacterial contamination. In contrast, ICU patients often have severe illness, prolonged hospitalization, invasive devices, and frequent exposure to broad-spectrum antibiotics, creating higher colonization pressure and a greater likelihood of MDRO contamination. Therefore, total bacterial burden and MDRO burden may represent related but distinct infection prevention challenges.

The bacterial load thresholds used in this study were based on relevant Chinese standards. International guidance on healthcare textiles emphasizes safe handling, hygienic laundering, separation of clean and contaminated textiles, and prevention of cross-contamination, but universally accepted quantitative thresholds for in-use medical textiles remain limited. Therefore, direct comparison across countries should be interpreted cautiously.

Several limitations should be acknowledged. First, this was a single-centre cross-sectional study, which limits the generalizability of the findings. Second, the number of MRSA and CRKP isolates was small, limiting the robustness of risk factor analysis and subgroup comparisons. Third, patient clinical isolates, healthcare worker hand samples and laundry-process samples were not collected, so direct transmission pathways could not be confirmed. Fourth, environmental variables such as humidity, temperature, textile material, washing frequency and storage conditions were not fully assessed. Fourth, because sampling was conducted only from April to August 2024, potential seasonal and environmental influences, including humidity, temperature fluctuations, and air-conditioning conditions, could not be fully evaluated. These factors may affect bacterial survival and persistence on textile surfaces. Future longitudinal studies covering different seasons and incorporating environmental monitoring are warranted. Fifth, freshly laundered unused textiles were not sampled as controls; therefore, we could not distinguish contamination acquired during clinical use from contamination introduced during laundering, storage, or transport. Future studies should include freshly laundered control textiles to better define the sources of contamination. Because enrichment culture was not performed, low-level MDRO contamination may have been underestimated, particularly when target organisms were present at low abundance or mixed with other background flora. Finally, the numbers of MRSA and CRKP isolates were relatively small, particularly for CRKP. Therefore, subgroup analyses and multivariable regression estimates for these organisms may be statistically unstable and should be interpreted as exploratory rather than confirmatory.

## Conclusion

This study demonstrated that medical textiles in hospital settings can be contaminated with clinically important MDROs, particularly CRAB in ICU environments. Genomic analysis revealed several closely related isolates and shared resistance gene profiles, suggesting possible local contamination or shared reservoirs. These findings support the inclusion of medical textiles in hospital environmental surveillance and infection prevention strategies. Future studies should combine longitudinal textile sampling, patient isolate comparison and laundry-process assessment to clarify the role of medical textiles in MDRO transmission.

## Data Availability

The datasets presented in this study can be found in online repositories. The names of the repository/repositories and accession number(s) can be found at: https://www.ncbi.nlm.nih.gov/genbank/, PRJNA1297944.
